# Aurantiamide acetate suppresses the growth of malignant gliomas *in vitro* and *in vivo* by inhibiting autophagic flux

**DOI:** 10.1111/jcmm.12498

**Published:** 2015-02-20

**Authors:** Yi Yang, Li-hui Zhang, Bing-xian Yang, Jin-kui Tian, Lin Zhang

**Affiliations:** a Department of Pharmacology, Hangzhou Key Laboratory of Medical Neurobiology, School of Medicine, Hangzhou Normal UniversityHangzhou, China; b College of Biomedical Engineering & Instrument Science, Zhejiang UniversityHangzhou, China

**Keywords:** malignant glioma cell, growth inhibition, cell death, autophagic flux, tumour-bearing mice

## Abstract

We aim to investigate the effect of aurantiamide acetate isolated from the aerial parts of *Clematis terniflora* DC against gliomas. Human malignant glioma U87 and U251 cells were incubated with different concentrations (0–100 μM) of aurantiamide acetate. Aurantiamide acetate greatly decreased the cell viability in a dose- and time-dependent manner. It induced moderate mitochondrial fragmentation and the loss of mitochondrial membrane potential. No significant difference was found in the alternation of other intracellular organelles, although F-actin structure was slightly disturbed. Apparent ultrastructure alternation with increased autophagosome and autolysosome accumulation was observed in aurantiamide acetate-treated cells. The expression of LC3-II was greatly up-regulated in cells exposed to aurantiamide acetate (*P* < 0.05 compared with control). The cytoplasmic accumulation of autophagosomes and autolysosomes induced by aurantiamide acetate treatment was confirmed by fluorescent reporter protein labelling. Administration of chloroquine (CQ), which inhibits the fusion step of autophagosomes, further increased the accumulation of autophagosomes in the cytoplasm of U87 cells. Autophagy inhibition by 3-methyladenine, Bafilomycin A1 or CQ had no influence on aurantiamide acetate-induced cytotoxicity, whereas autophagy stimulator rapamycin significantly suppressed aurantiamide acetate-induced cell death. The anti-tumour effects of aurantiamide acetate were further evaluated in tumour-bearing nude mice. Intratumoural injection of aurantiamide acetate obviously suppressed tumour growth, and increased number of autophagic vacuoles was observed in tumour tissues of animals receiving aurantiamide acetate. Our findings suggest that aurantiamide acetate may suppress the growth of malignant gliomas by blocking autophagic flux.

## Introduction

Gliomas are the most aggressive brain malignancies and are associated with high mortality 1. Despite considerable advances having been achieved in the treatment of high-grade glioma, including surgery, irradiation, chemotherapy and even gene therapy, the prognosis is still poor possibly because of the tumour heterogeneity and strong therapeutic resistance 2. Therefore, novel therapeutic strategies are urgently needed.

*Clematis* has been recognized as a botanical source for diverse pharmaceutically active components 3. *Clematis terniflora* DC is mainly distributed in eastern and south-western Asia, northern Africa, Europe 4, and the aerial part has long been used as Chinese folk medicine in the treatment of urinary infections as well as brain tumour. Our previous study demonstrated that the extracts of the aerial part of *C. terniflora* DC had anti-inflammatory and antinociceptive activities in rat model of carrageenan-induced chronic non-bacterial prostatitis (CNP) 5. Nevertheless, the molecular mechanism of the therapeutic effects of *C. terniflora* DC on human malignant glioma has not yet been reported.

Macroautophagy (hereafter referred to as autophagy) is a well-characterized ‘self-eating’ process, which involves the initial sequestration of cytoplasmic materials in double-membraned autophagosomes followed by degradation of encapsulated components or cargos after fusion with lysosomes. In many human diseases, autophagy acts as a double-edged sword. Insufficient autophagy or excessive accumulation of autophagosomes and autolysosomes caused by disturbed autophagic flux both contribute to cell death 6,7. Therefore, autophagy modulation represents a promising target for new therapies for human disorders, including cancer 8. It has been indicated that malignant glioma cell death induced by a variety of anti-tumour reagents, such as temozolomide 9, arsenic trioxide 10,11, curcumin 12, is associated with increased autophagic activity. In contrast, blockade of the basal autophagic flux also induces glioma cell death 13.

In this study, we isolated aurantiamide acetate from the aerial parts of *C. terniflora* DC and investigated the *in vitro* and *in vivo* anti-tumour effects of aurantiamide acetate on human malignant glioma cells. Our findings may provide valuable insights into understanding the mechanism of aurantiamide acetate-mediated tumour growth arrest and implicate that aurantiamide acetate may be a promising compound for the therapy of brain tumours.

## Materials and methods

### Reagents

Aurantiamide acetate was isolated from the aerial parts of *C. terniflora* DC in our laboratory. Cisplatin was provided by QiLu Pharmaceutical (Jinan, China). Bafilomycin A1 (BafA1; BML-CM110-0100) and rapamycin (BML-A275-0005) were obtained from Alexis Biochemicals (Lausen, Switzerland). 3-methyladenine (M9281) and chloroquine (CQ; C6628) were bought from Sigma-Aldrich (Shanghai) Trading Co., Ltd, Shanghai, China. EYFP (enhanced yellow fluorescence protein)-Golgi plasmid was purchased from Clontech Laboratories, Inc., Mountain View, CA, USA. MitoTracker Red CMXRos and propidium iodide (PI) were from Invitrogen Life Technologies, CA, USA Carlsbad. Alexa Fluor 488 Phalloidin was obtained from Molecular Probes, Inc., Eugene, OR, USA. DMEM and heat-inactivated horse serum were obtained from GIBCO-BRL, New York, NY, USA. Foetal calf serum (FCS) was purchased from Hangzhou Tianhang Biological Technology Co., Ltd., Hangzhou, China. Anti-LC3 and anti-actin primary antibody was purchased from Cell Signaling Technology Shanghai Biological Reagents Co., Ltd., Shanghai, China, and Santa Cruz Biotechnology (Shanghai) Co., Ltd., China respectively.

### Preparation of aurantiamide acetate

Aurantiamide acetate was isolated from the aerial parts of *C. terniflora* DC in our laboratory. In brief, the fresh stems and leaves of *C. terniflora* DC were dried at room temperature and cut into small pieces. Samples (10 kg) were extracted by 70% ethanol and the ethanol extract was concentrated by vacuum evaporation, resolved in water, purified by AB-8 macroporous resin, and eluted with 70% ethanol. Approximately 250 g ethanol extract was obtained and the overall yield was 2.5%. Then the ethanol extract of *C. terniflora* DC (100 g) was partitioned with silica gel column chromatography with chloroform–methanol mixed solvent system. The fraction (chloroform: methanol = 98: 2; v/v) was purified using gel permeation chromatography on a Sephadex LH-20 column, eluted with 50% chloroform and methanol solvents, to give 960 mg aurantiamide acetate. High-performance liquid chromatography (HPLC) chromatograms of *C. terniflora* DC ethanol extract and aurantiamide acetate were shown in Figure S1. The purification of aurantiamide acetate was 98%. The structure of aurantiamide acetate was presented in Figure S2.

### Cell culture

Human malignant glioma U251 cells, human hepatoma HepG2 cells and highly differentiated rat PC12 pheochromocytoma cells were purchased from the Shanghai Cell Bank, Chinese Academy of Sciences (Shanghai, China). Human malignant glioma U87 cells were obtained from American Type Culture Collection (Rockville, MD, USA). U87, U251 and HepG2 cells were cultured in DMEM supplemented with 5% FCS, 50 U/ml penicillin and 50 μg/ml streptomycin. PC12 cells were cultured in DMEM supplemented with 5% FCS, 10% heat-inactivated horse serum, 50 U/ml penicillin and 50 μg/ml streptomycin. Cell cultures were maintained at 37°C and 5% CO_2_ in a humidified environment.

### Determination of cell viability

Cell viability was evaluated using MTT assay 14. Briefly, cells were seeded into 96-well plates at a density of 500 cells/well (low density) or 1500 cells/well (high density). Twenty-four hours after initial seeding, cells were treated with different concentrations of aurantiamide acetate, ranging from 0 to 100 μM. After the appropriate incubation time, 15% volume of MTT (5 mg/ml) was added to each well. Cells were incubated for another 4 hrs at 37°C. Then, the medium was removed and 100 μl dimethyl sulfoxide was added to each well to resuspend the MTT metabolic product. The absorbance of the dissolved formazan was measured at 570 nm (A570) with a microplate reader (Versa Max; Molecular Devices Sunnyvale, CA, USA). Each treatment was performed in sextuplicate and data were calculated from three independent experiments. The percentage of cell proliferation was calculated by the following formula: Cell proliferation (%) = 100 − [(*A*_570, Control_ − *A*_570, Sample_)/*A*_570, Control_] × 100.

### Hoechst 33342 staining

After 48-hrs of aurantiamide acetate (25 μM) or cisplatin (2.5 μg/ml) incubation, cells were fixed with 4% paraformaldehyde (PFA) for 20 min. at room temperature, then washed three times with PBS and exposed to 10 mg/l Hoechst 33342 at 37°C in dark for 15 min. After PBS washing, samples were observed under fluorescence microscopy (Olympus IX81; Olympus, Tokyo, Japan; 60×; NA = 1.45). At least 20 photomicrographs were evaluated randomly per treatment and representative data were presented.

### Transfection

Transfection was performed as described previously 15. For each well in a 24-well plate, 1 μg DNA plasmid was combined with 2 μl lipofectamine 2000 in serum-free DMEM. Mixture was incubated at room temperature for 20 min., and another 200 μl serum-free DMEM was added. Cell cultures were left for 1 hr in this transfection medium and then it was replaced with fresh culture medium. Fluorescence was detected under fluorescence microscopy (Olympus IX81; 60×; NA = 1.45) or Zeiss Laser Scanning Confocal Microscope (Zeiss Germany LSM 510; 100×; NA = 1.30; Zeiss LSM 510; (Zeiss LSM 510; 100×; NA = 1.30; Carl Zeiss Inc., Jena, Germany).

### MitoTracker staining

MitoTracker staining was performed as described previously 16,17. In brief, cells were incubated with 100 nM Mitotracker Red CMXRos at 37°C in dark for 20 min. After three times washing with DMEM, the fluorescence was detected under fluorescence microscopy (Olympus IX81; 60×; NA = 1.45). The mean fluorescence intensity was measured by ImageJ 1.47e software (Wayne Rasband, National Institutes of Health, Bethesda, MD, USA).

### Determination of actin filaments

Cells were fixed with 4% PFA, permeabilized with PBS containing 0.3% Triton X-100 and 10% goat serum, and incubated with Alexa Fluor 488 Phalloidin. After three times washing with PBS, the nuclei were counterstained with PI for another 10 min. After washing, the fluorescence was detected under laser scanning confocal microscope (Zeiss LSM 510; 100×; NA = 1.30).

### Transmission electron microscopy analysis

Transmission electron microscopy (TEM) analysis was conducted as reported 18,19. The cells or tissue samples were washed twice with PBS, and fixed in 2.5% glutaraldehyde solution for 2 hrs and postfixed in 1% osmium tetroxide solution for 1 hr. After dehydration in an ethanol gradient, samples were incubated with propylenoxide, impregnated with a mixture of propylenoid/LX-112 and embedded in LX-112. Ultrathin sections were stained with uranyl acetate and lead citrate, and examined in a 100CX-II TEM (Jeol, Tokyo, Japan) operating at 80 kV.

### Western blotting

Protein expression was evaluated by Western blotting as reported previously 20. Briefly, total protein was extracted from cells and the protein concentration was measured using bicinchoninic acid protein assay kit (Beyotime Institute of Biotechnology, Haimen, Jiangsu, China). Equal amounts of protein extracts were separated by 12% SDS-PAGE, transferred to a PVDF membrane (Millipore, Billerica, MA, USA), blocked with 5% w/v non-fat dry milk dissolved in Tris buffered saline plus Tween-20 (TBS-T; 0.1% Tween-20), and incubated with anti-LC3 primary antibody (1:2000 dilution) at 4°C overnight. After washing with TBS-T, membranes were probed with horseradish peroxidase (HRP)-conjugated secondary antibody (1:2000 dilution) for 2 hrs at room temperature. Immunobands were visualized using enhanced chemiluminescence kit. The expression bands of target proteins were analysed by Photoshop software, and the densitometric values were used to conduct statistical analysis. The housekeeping protein actin was used as an internal control.

### Animal studies

Animal studies were conducted as described previously 12 with minor modifications. Six-week-old nude mice (five mice per treatment group), weighing approximately 20 g, were provided by Experimental Animal Center of Zhejiang Academy of Medical Sciences (Hangzhou, China). U87 cells (1 × 10^6^ cells in 100 μl serum-free DMEM) were inoculated subcutaneously into the right axillary region of the flank of nude mice. Tumour growth was measured with callipers and animal weight was recorded each week. Tumour volume was calculated as (4π/3) × (width/2)^2^ × (length/2). Three weeks following tumour cell inoculation, the tumours reached an average volume of 200 mm^3^. Then, a 150 μl intratumoural injection of aurantiamide acetate (1 mg in PBS) or the same volume of PBS was given every 2 days. The day of initial drug administration was recognized as Day 0. Drug administration was continued for 8 days and the tumour size was measured on Day 10. After measurement, mice were killed and tumours were carefully removed and weighed. Some of the tumour tissues were used for electron microscopy analysis. All procedures were conducted in accordance with the standards promulgated by Decree No. 2 of the State Science and Technology Commission in China. The animal experiments were approved by Hangzhou Normal University.

### Statistical analysis

Data were analysed using SPSS16.0 IBM Corporation, Armonk, NY, USA software and were plotted as mean ± SEM. Statistically significant differences were carried out by one-way anova, followed by Tukey's *post hoc* tests. Asterisks denoted statistical significance, with *P* < 0.05.

## Results

### Cytotoxic activity of aurantiamide acetate in cell culture

**Figure 1:**
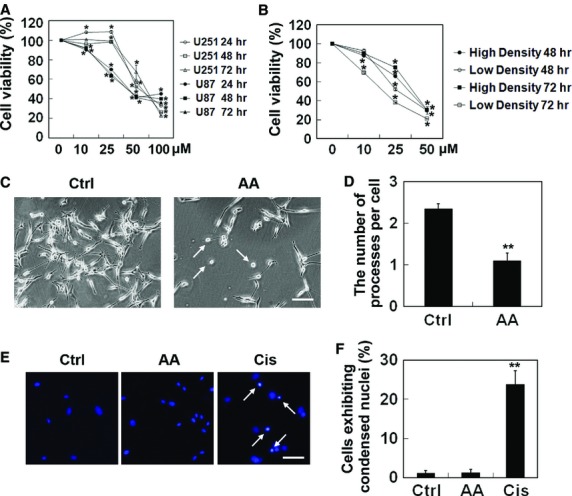
Cytotoxic activity of aurantiamide acetate on *in vitro* cultured human malignant glioma U87 and U251. (A) U251 and U87 cells were seeded into 96-well plates at a density of 1000 cells/well. Twenty-four hours after initial seeding, cells were treated with different concentrations of aurantiamide acetate (0, 10, 25, 50 or 100 μM). Following different time period (0, 24, 48 or 72 hrs) of drug incubation, the cell viability was determined by MTT. (B) U87 cells were seeded into 96-well plates at a density of 500 cells/well (low density) or 1500 cells/well (high density). Twenty-four hours after initial seeding, cells were incubated with aurantiamide acetate (0, 10, 25 or 50 μM). Following different time period (0, 48 or 72 hrs) of drug incubation, the cell viability was determined by MTT. (C) Representative cellular morphology after 48-hrs incubation of 25 μM aurantiamide acetate (AA). Dead cells were indicated by arrows. (D) The number of processes per cell was quantified from six micrographs. At least 30 cells per micrograph were analysed. (E) Cells treated with 25 μM AA or 2.5 μg/ml cisplatin (Cis) for 48 hrs were stained with Hoechst 33342. Apoptotic cells were indicated by arrows; scale bars, 50 μm. (F) The proportion of cells exhibiting condensed apoptotic nuclei was calculated from 20 micrographs. *P < 0.05; **P < 0.01 compared with control.

The cytotoxic activity of aurantiamide acetate was determined by MTT on *in vitro* cultured human malignant glioma U87 and U251 cells over a period of 72 hrs. As revealed by Figure1A, aurantiamide acetate decreased the cell viability in a dose- and time-dependent manner. After 24-hrs incubation of 10 μM aurantiamide acetate, the viability of U87 cells was greatly reduced (*P* < 0.05 compared with control). Prolonged incubation time or elevated dose of drug decreased cell viability. Compared with U251 cells, U87 cells appeared to be more sensitive upon aurantiamide acetate treatment. In addition, the degree of cytotoxicity observed with aurantiamide acetate seemed to depend on the initial seeding density of U87 cells. Cells with low density (500 cells/well) appeared to be more vulnerable to aurantiamide acetate-induced cytotoxicity as compared with those with high density (1500 cells/well; Fig.1B). Considering that 48-hrs of 25 μM aurantiamide acetate incubation significantly suppressed cell viability of U87 cells to approximately 60%, such dose and incubation period were used for the following experiments. Note that not all cells were sensitive to aurantiamide acetate, as 25 μM drug incubation for 48 hrs had no significant influence on the cell viability of PC12 pheochromocytoma cells (Fig. S3A). Aurantiamide acetate at the dose of 10–50 μM induced a 20% reduction in the cell viability in human hepatoma HepG2 cells after 48-hrs incubation (Fig. S3A). These data suggest that cells have different response to aurantiamide acetate incubation.

### Aurantiamide acetate did not induce apoptosis in U87 cells

Under bright field microscope, the normal U87 cells showed multiple processes extended from their cell bodies (Fig.1C). After exposed to aurantiamide acetate for 48 hrs, the number of the cells was decreased. Some of the cells became shrunk and even detached from culture dish. The number of processes extended from cells was also decreased (*P* < 0.01 compared with control; Fig.1D). To understand the underlying mechanism of aurantiamide acetate-induced cytotoxicity in U87 cells, Hoechst 33342 staining was used to detect the apoptotic changes in cells treated with aurantiamide acetate. The nuclei of non-treated or aurantiamide acetate-treated cells stained uniformly with Hoechst 33342, indicating that no cell apoptosis occurred in these cells (Fig.1E and F). Positive control cells were treated with cisplatin (2.5 μg/ml) for 48 hrs, and significantly increased number of cells exhibiting condensed apoptotic nuclei was detected in this group (Fig.1E and F). These data demonstrate that apoptosis may not be involved in aurantiamide acetate-induced cytotoxicity in U87 cells.

### Influence of aurantiamide acetate on intracellular organelles of U87 cells

**Figure 2:**
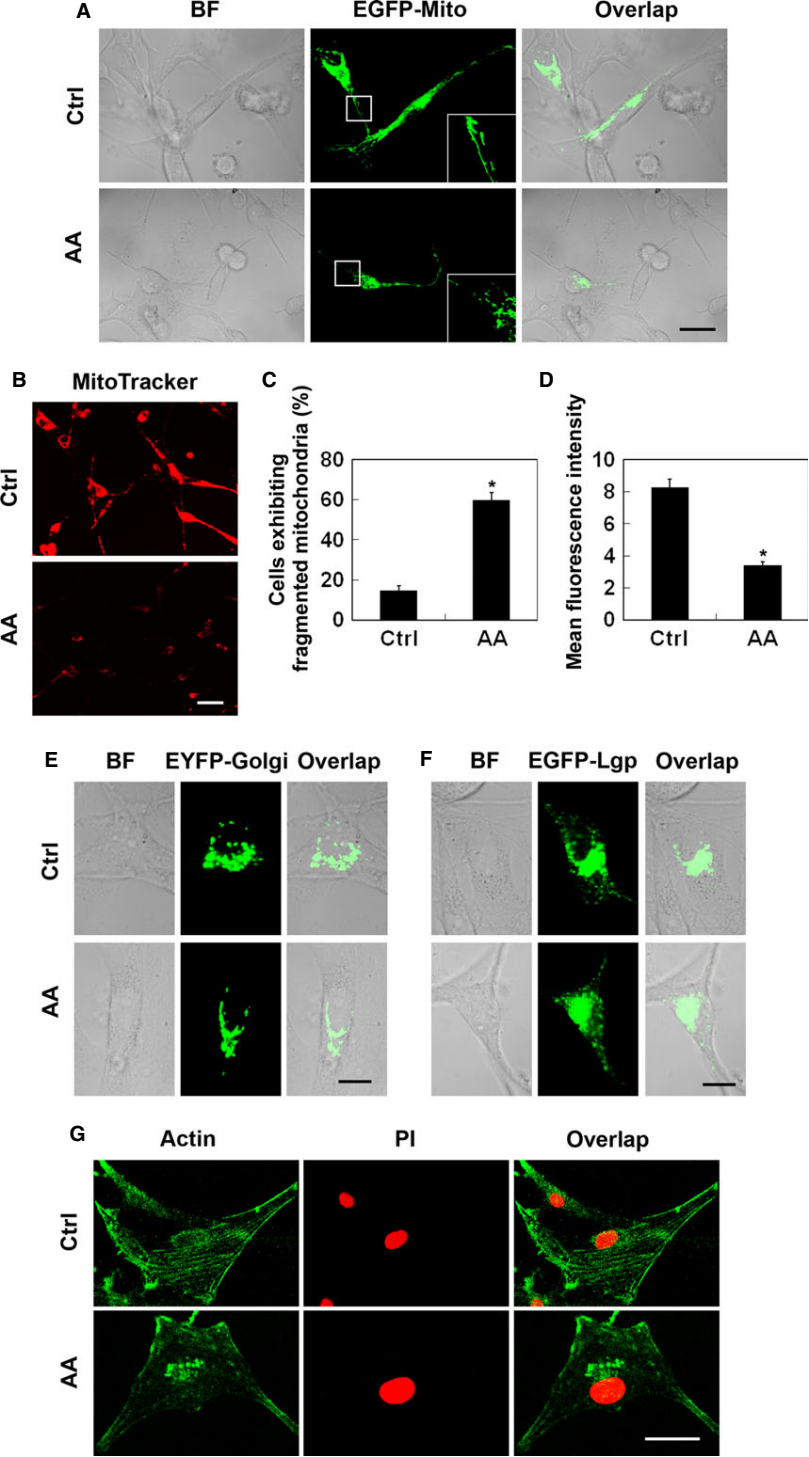
Influence of aurantiamide acetate on intracellular organelles of U87 cells. Cells were transfected with EGFP-Mito (A), EYFP-Golgi (E) or EGFP-Lgp120 (F) to visualize the alternations of mitochondria, Golgi apparatus or lysosomes. Some of the cells were stained with MitoTracker Red (B–D) to determine mitochondrial membrane potential or co-stained with Phalloidin and PI (G) to label F-actin and nucleus respectively. Normal healthy U87 cells (Ctrl) or cells following 48-hrs of 25 μM aurantiamide acetate incubation (AA) were used for analysis of the intracellular organelle alternation. Boxed area in (A) was enlarged in the same figure. BF, bright field. Scale bars in (A), (B) and (G), 20 μm; scale bars in (E) and (F), 10 μm. Quantified data were shown in (C) and (D). Data were calculated from 12 micrographs. *P < 0.05 compared with control.

To understand aurantiamide acetate-induced cytotoxicity, we examined the alternations of intracellular organelles using live cell imaging and immunofluorescence analysis. Healthy U87 cells pre-transfected with EGFP-Mito plasmid contained numerous rod-like or filamentous-shaped mitochondria (Fig.2A). Aurantiamide acetate treatment increased the proportion of fragmented mitochondria, and the loss of mitochondrial membrane potential was also detected in cells exposed to aurantiamide acetate (Fig.2B–D). No significant difference was observed in the number or distribution of Golgi apparatus (labelled with EYFP-Golgi) or lysosomes [labelled with EGFP-lysosomal glycoprotein (Lgp) 120] between control and drug-treated cells (Fig.2E and F). Golgi apparatus and lysosomes in both groups located in the perinuclear region of the cells. Phalloidin staining indicated that the actin filaments arranged regularly in the cytoplasm of normal cells. However, the F-actin structure was slightly disturbed in cells incubated with aurantiamide acetate, as long filaments were invisible in the cytoplasm (Fig.2G).

### Aurantiamide acetate-induced autophagosome and autolysosome accumulation in U87 cells

**Figure 3:**
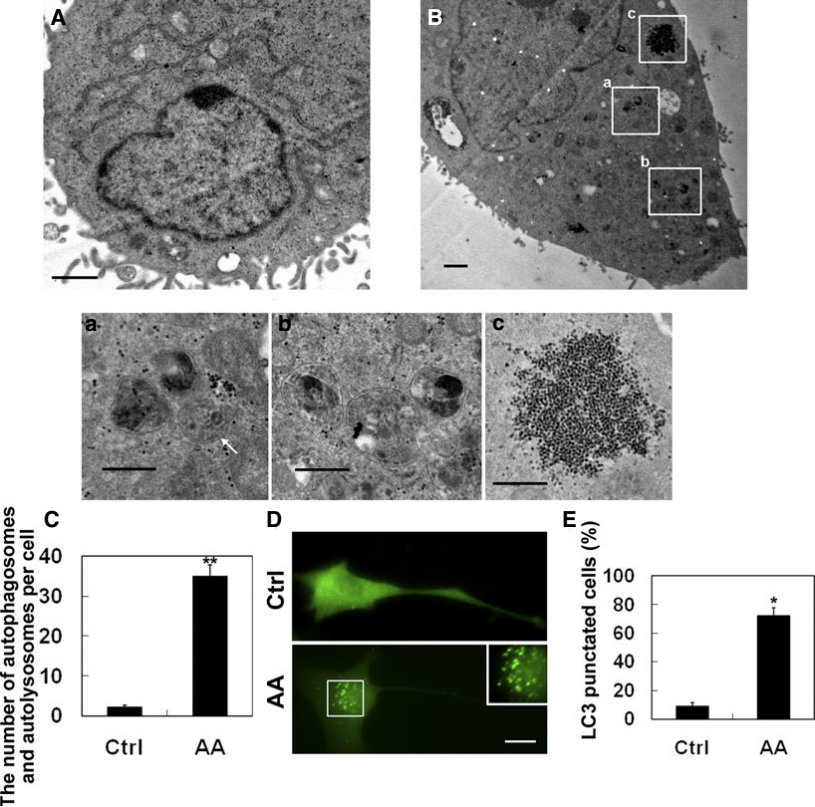
Aurantiamide acetate-induced autophagosome and autolysosome accumulation in U87 cells. Normal healthy U87 cells (A) or cells following 48-hrs of 25 μM aurantiamide acetate incubation (B) were fixed. The ultrastructure alternations were observed under TEM. Boxed areas in (B) were enlarged in (a–c). (a) The *de novo* formation of double membrane-bound phagophore was indicated by arrow. (b) Autophagosomes engulfed with material to be degraded. (c) Glycogen particles. Scale bars in (A) and (B), 1 μm; scale bars in (a–c), 0.5 μm. (C) The number of autophagosomes and autolysosomes per cell was calculated from 12 micrographs. (D and E) U87 cells were transfected with GFP-LC3 plasmid. Following 48-hrs of 25 μM AA incubation, the fluorescence was examined under fluorescent microscope. Inset shows enlarged image of boxed region. (E) The percentage of LC3 punctated cells was calculated from 12 micrographs. *P < 0.05; **P < 0.01 compared with control.

We next investigated the ultrastructure alternations of U87 cells following aurantiamide acetate exposure. As shown by Figure3A, healthy U87 cells exhibited normal ultrastructure with intact nucleus, cytoplasm and intracellular organelles. However, after aurantiamide acetate treatment, abundant single- and double-membrane bounded structures, known as autophagosomes and autolysosomes, were observed within cytoplasm (Fig.3B). In a few samples, we observed the *de novo* formation of double membrane-bound phagophore (Fig.3Ba). Most of the autophagosomes engulfed with material to be degraded (Fig.3Ba, b). Besides, plenty of glycogen particles were also found in cells exposed to aurantiamide acetate (Fig.3Bc). Statistical analysis showed that the number of autophagosomes and autolysosomes per cell was significantly elevated in aurantiamide acetate-treated cells (*P* < 0.01 compared with control; Fig.3C). To visualize the intracellular distribution of autophagosomes in living cells, U87 cells were transfected with green fluorescence protein-light chain 3 (GFP-LC3). Under basal condition, green fluorescence was uniformly distributed in U87 cells (Fig.3D). After aurantiamide acetate incubation, the accumulation of GFP-LC3-labelled autophagosomes was observed mainly in perinuclear regions (Fig.3D). The percentage of cells with LC3-positive vesicles was significantly increased in aurantiamide acetate-treated cells (*P* < 0.01 compared with control; Fig.3E). The increased accumulation of autophagosomes and autolysosomes in cells treated by aurantiamide acetate suggests that aurantiamide acetate may either trigger the increased autophagic activity or inhibit autophagic/lysosomal protein degradation in U87 cells.

### Aurantiamide acetate impaired autophagic flux in U87 cells

**Figure 4:**
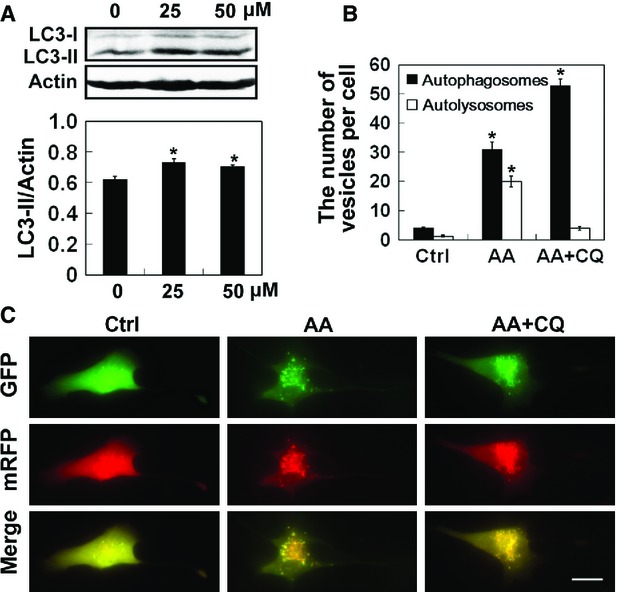
Autophagy flux in cells exposed to aurantiamide acetate. (A) U87 cells were incubated with 0, 25 or 50 μM aurantiamide acetate (AA). The expressions of LC3-I and LC3-II in cells were examined by Western blotting. Actin was used as internal control. Data were quantified from three independent experiments. (B and C) U87 cells transfected with mRFP-GFP-LC3 were exposed to AA or AA plus 10 μM chloroquine (AA+CQ). The fluorescence was examined under fluorescent microscope. GFP (sensitive to acidification and lysosomal degradation)-labelled vesicles were autophagosomes; mRFP (insensitive to acidification and lysosomal degradation)-labelled vesicles were autophagosomes and autolysosomes; vesicles expressing only mRFP were autolysosomes. (G) The number of autophagosomes and autolysosomes per cell was calculated; scale bars, 20 μm. *P < 0.05 compared with control.

To understand the effects of aurantiamide acetate on autophagic flux, the LC3-II conversion in U87 cells was studied using Western blotting. We found that the level of LC3-I (cytosolic) isoform was not greatly altered, while the expression of LC3-II (autophagosome associated) isoform was significantly elevated after cells were incubated with 25 or 50 μM aurantiamide acetate for 48 hrs (*P* < 0.05 compared with control; Fig.4A). The monomeric red fluorescence protein-green fluorescence protein-light chain 3 (mRFP-GFP-LC3) reporter construct is widely used to measure the autophagosome turnover, in which GFP fluorescence was more sensitive to acidic pH after fusion with lysosomes 21. Here, U87 cells were transfected with mRFP-GFP-LC3 plasmid, in which green signals indicate autophagosomes and red signals indicate autophagosomes as well as autolysosomes. Likewise, few autophagosomes and autolysosomes were detected in normal U87 cells expressing mRFP-GFP-LC3 (Fig.4B). The number of autophagosomes and autolysosomes was greatly increased upon aurantiamide acetate treatment, and a great portion of these vesicles was autolysosomes (Fig.4C). Administration of CQ, a lysosomotropic agent that inhibits the fusion step of autophagosomes, increased the accumulation of autophagosomes in the cytoplasm of U87 cells, while the overall number of autophagosomes and autolysosomes was not greatly changed. These findings indicate that the aurantiamide acetate may inhibit tumour cell growth by suppressing the autophagic/lysosomal protein degradation. Aurantiamide acetate treatment slightly increased the LC3-II level in PC12 cells and induced the formation of large puncta within cytoplasm of PC12 cells. Although aurantiamide acetate elevated the number of autophagosomes in both HepG2 and PC12 cells, no significant difference was found either in the LC3-II conversion or the number of autophagosomes per cell (Fig. S3B–F), implying that the changes in aurantiamide acetate-induced growth inhibition might be related to the varied autophagic flux in different cell types exposed to aurantiamide acetate.

### Influence of autophagy interference on aurantiamide acetate-induced cell death

**Figure 5:**
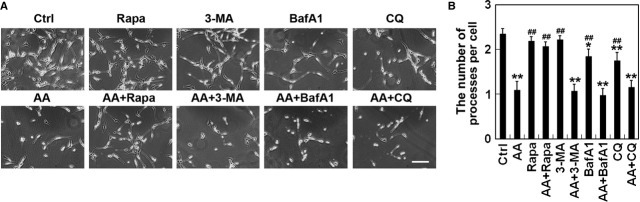
Influence of autophagy stimulator or inhibitors on aurantiamide acetate-induced cell death. U87 cells were incubated with 25 μM aurantiamide acetate (AA), 10 nM rapamycin (Rapa), 10 mM 3-methyladenine (3-MA), 40 nM Bafilomycin A1 (BafA1), 10 μM chloroquine (CQ), 25 μM AA plus 10 nM Rapa, 25 μM AA plus 10 mM 3-MA, 25 μM AA plus 40 nM BafA1, or 25 μM AA plus 10 μM CQ. Cells without drug treatment were used as control (Ctrl). (A) Representative images were presented; scale bar, 50 μm. (B) The number of processes per cell was quantified from six micrographs. At least 30 cells per micrograph were analysed. *P < 0.05; **P < 0.01 compared with control; ##*P* < 0.01 compared with AA.

To understand the role of autophagy in aurantiamide acetate-induced cell death, autophagy stimulator and inhibitors were used. As revealed by Figure5A, no significant difference was found in cell morphology treated with rapamycin, 3-MA, BafA1 or CQ. However, BafA1 or CQ administration reduced the number of processes in cells (*P* < 0.05 compared with control; Fig.5B). Aurantiamide acetate incubation dramatically induced cytotoxicity, with the appearance of suppressed cell growth and reduced number of processes per cell (*P* < 0.05 compared with control). In addition, autophagy inhibition by using 3-MA, BafA1 or CQ had no protective effects on aurantiamide acetate-induced cytotoxicity (*P* > 0.05 compared with aurantiamide acetate alone). Administration of autophagy stimulator rapamycin significantly suppressed aurantiamide acetate-induced loss of processes (*P* < 0.05 compared with aurantiamide acetate alone), suggesting that the insufficient autophagic clearance caused by impaired autophagic turnover may contribute to the aurantiamide acetate-induced U87 cell death.

### Aurantiamide acetate inhibited growth of subcutaneous tumours

**Figure 6:**
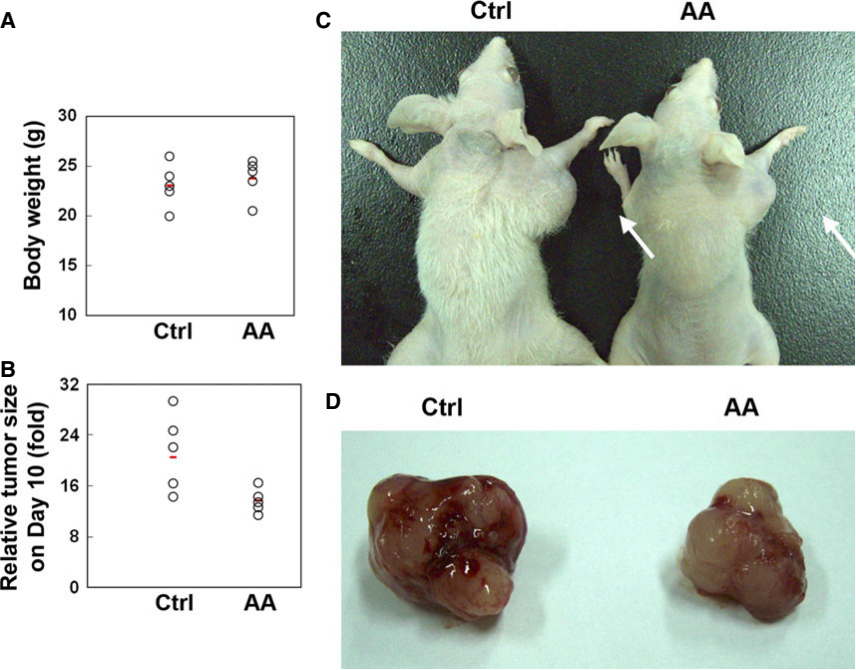
Aurantiamide acetate inhibited the growth of subcutaneous tumours. U87 cells were inoculated subcutaneously into the right axillary region of the flank of nude mice. Three weeks following inoculation, a 150 μl intratumoural injection of aurantiamide acetate (AA) or the same volume of PBS (Ctrl) was given every 2 days. Drug administration was lasted for 8 days and the tumour size was measured on Day 10. Data were calculated from five animals for each group. (A) Animal body weight (g). (B) Relative tumour size on Day 10. Representative data of gross examination of tumour-bearing mice (C) and tumour tissues (D).

**Figure 7:**
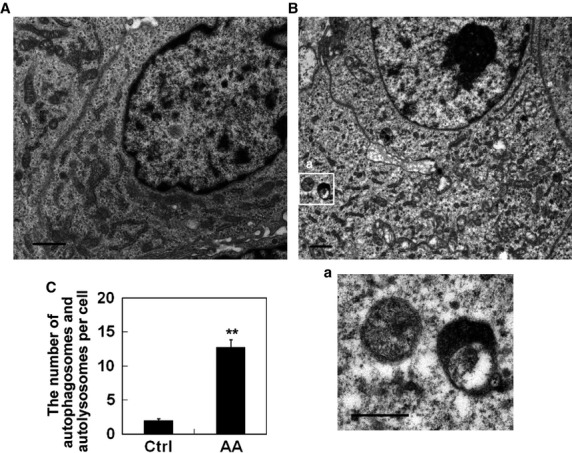
TEM analysis of tumour tissues derived from tumour-bearing nude mice. Tumours were removed from killed animals on Day 10 after drug administration. (A) Tumour section derived from control animal. (B) Tumour section derived from animals received intratumoural injection of aurantiamide acetate. Boxed areas in (B) were enlarged in (a). *N* = 5 for each group. Scale bars in (A) and (B), 1 μm; scale bar in (a), 0.5 μm. (C) The number of autophagosomes and autolysosomes per cell was calculated from 12 micrographs. **P < 0.01 compared with control.

The *in vivo* anti-tumour effect of aurantiamide acetate was evaluated using tumour-bearing nude mice. As shown by Figure6A, the intratumoural injection of aurantiamide acetate administration had no significant influence on the animal body weight as compared with control (*P* > 0.05). However, aurantiamide acetate obviously decreased the growth of tumour size (*P* < 0.05; Fig.6B–D). TEM analysis of tumour tissues derived from tumour-bearing mice further demonstrated that a significantly elevated number of autophagosomes and autolysosomes in the tumour tissues received aurantiamide acetate injection (Fig.7A–C).

## Discussion

*Clematis terniflora* DC is widely distributed in Zhejiang Province, China, and is used as a Chinese herbal medicine for a long historical tradition. The aerial part has been used as Chinese folk medicine for the treatment of brain tumour, although the molecular mechanism of the anti-tumour effects of *C. terniflora* DC is not yet illustrated. In our previous study, we isolated and identified extracts from the aerial part of *C. terniflora* DC and indicated that these extracts had anti-inflammatory and antinociceptive activities in a rodent model 5. Here, we observed that the aurantiamide acetate derived from *C. terniflora* DC had the ability to suppress the *in vitro* and *in vivo* growth of human malignant gliomas and assessed the underlying mechanisms involved in aurantiamide acetate-mediated anti-tumour action.

In our preliminary studies, the cytotoxicity of aurantiamide acetate was evaluated using several different cell lines maintained in our laboratory, including human malignant glioma U251, U87 cells, human liver hepatocellular carcinoma HepG2 cells, and rat pheochromocytoma PC12 cells. We found that human malignant glioma U251 and U87 cells, especially U87 cells, were the most sensitive cell line that responded to the treatment of aurantiamide acetate, implying that aurantiamide acetate may specifically suppress the growth of malignant gliomas, but not other tumour cells. Otherwise, it is possible that other cells might be more resistant to aurantiamide acetate. Indeed, *in vitro* and *in vivo* studies confirmed the glioma growth arresting activities of aurantiamide acetate. A 48-hrs incubation of 25 μM aurantiamide acetate significantly suppressed cell viability of U87 cells to approximately 60%, and intratumoural injection of aurantiamide acetate greatly reduced the tumour size in tumour-bearing nude mice. After 48-hrs of aurantiamide acetate incubation, the 50% inhibitory concentration was approximately 50 μM. It is reported that 72-hrs of curcumin incubation also decreased the cell viability of U87 cells, with a 50% inhibitory concentration of 35 μM 12. Besides, treatment with arsenic trioxide (2 or 4 μM) for 96 hrs not only inhibited cell viability but also induced cell death in U87 cells 10. The 50% inhibitory concentration of temozolomide for U87 cells was less than 200 μM after 72-hrs incubation 9. Therefore, aurantiamide acetate appears to be a promising agent for human malignant glioma therapy, and future study will be continued to explore the potential effects of combined application of aurantiamide acetate with one or more anti-glioma agents on tumour growth arresting.

Accumulating evidence suggests that many anti-glioma agents, such as temozolomide 9, arsenic trioxide 11, curcumin 12, and C_2_-ceramide 22, may induce non-apoptotic cell death in *in vitro* cultured malignant glioma cells. Instead, autophagy plays an essential role in drugs-mediated cell death. In this study, apoptotic cell death was not found in aurantiamide acetate-treated cells, while we detected a significantly increased number of double-membraned autophagosomes and single-membraned autolysosomes in the cytoplasm of U87 cells upon aurantiamide acetate incubation. Autophagic cell death is identified when cell death meets the following criteria: During autophagic cell death, the cell death may occur without the apoptosis induction, but is accompanied by an elevated autophagic flux 23. Importantly, pharmacological or genetic inhibition of autophagy prevents cell death 23. In our present study, autophagy inhibition by 10 mM 3-methyladenine, 40 nM Baf A1 or 10 μM CQ did not abolish aurantiamide acetate-induced cell death. However, 10 nM rapamycin administration protected against cell death induced by aurantiamide acetate. These findings reveal that aurantiamide acetate may not induce autophagic cell death. In contrast, it is possible that the accumulation of autophagosomes and autolysosomes may reflect an impairment of autophagic flux. As shown by Western blotting analysis, the LC3-I expression was not greatly altered in cells after aurantiamide acetate incubation, whereas LC3-II level was significantly up-regulated, indicating that aurantiamide acetate might inhibit autolysosome degradation rather than autophagy stimulation. The accumulation of autophagosomes and autolysosomes may delay the intracellular clearance of damaged proteins and organelles. Therefore, autophagy stimulation by rapamycin prevented cell death. Isshiki *et al*. reported that aurantiamide acetate was a selective cathepsin inhibitor, which inhibited cysteine proteinases, particularly cathepsin L and B 24. Based on this evidence, we speculate that aurantiamide acetate may induce the accumulation of autophagosomes and autolysosomes by inhibiting the degradation of lysosomal cysteine proteinases. Future study will focus on the precise role of autophagy in aurantiamide acetate-mediated cell death. Also, we could not exclude the possibility that other mechanisms, such as necrosis, might be involved in aurantiamide acetate-mediated anti-tumour activities.

It should also be noted that mitochondrial damage might be involved in the aurantiamide acetate-induced autophagy, as a significant loss of mitochondrial membrane potential as well as increased mitochondrial fragmentation was observed in U87 cells treated with aurantiamide acetate. In accordance with our findings, arsenic trioxide has been shown to cause autophagic cell death in glioma cells by disrupting the mitochondrial membrane integrity 9. In our future study, we will focus on exploring the mechanism of mitochondrial dysfunction in aurantiamide acetate-induced autophagy in glioma cells.

In summary, our *in vitro* and *in vivo* studies demonstrate that aurantiamide acetate may suppress the growth of human malignant gliomas *via* inhibiting intracellular autophagic flux. Aurantiamide acetate may be a promising compound for the therapy of malignant gliomas.

## Supporting Information

**Figure S1** HPLC chromatograms.

**Figure S2** Chemical structure of aurantiamide acetate.

**Figure S3** Effects of aurantiamide acetate on HepG2 and PC12 cells.
