# Supplementary figures and images for: Aurantiamide acetate suppresses the growth of malignant gliomas in vitro and in vivo by inhibiting autophagic flux
Source: J Cell Mol Med. 2015 Feb 20;19(5):1055–64. doi: 10.1111/jcmm.12498 (PMC4420607; doi:10.1111/jcmm.12498)

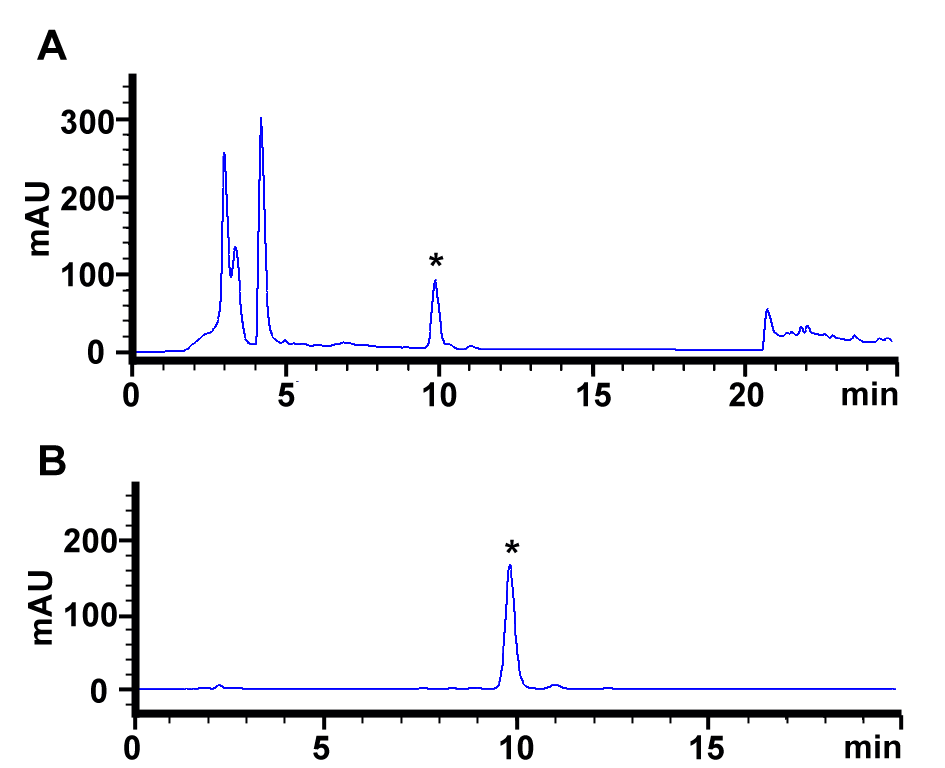

Supplement: Supplementary file 1 [file jcmm0019-1055-sd1.tif]

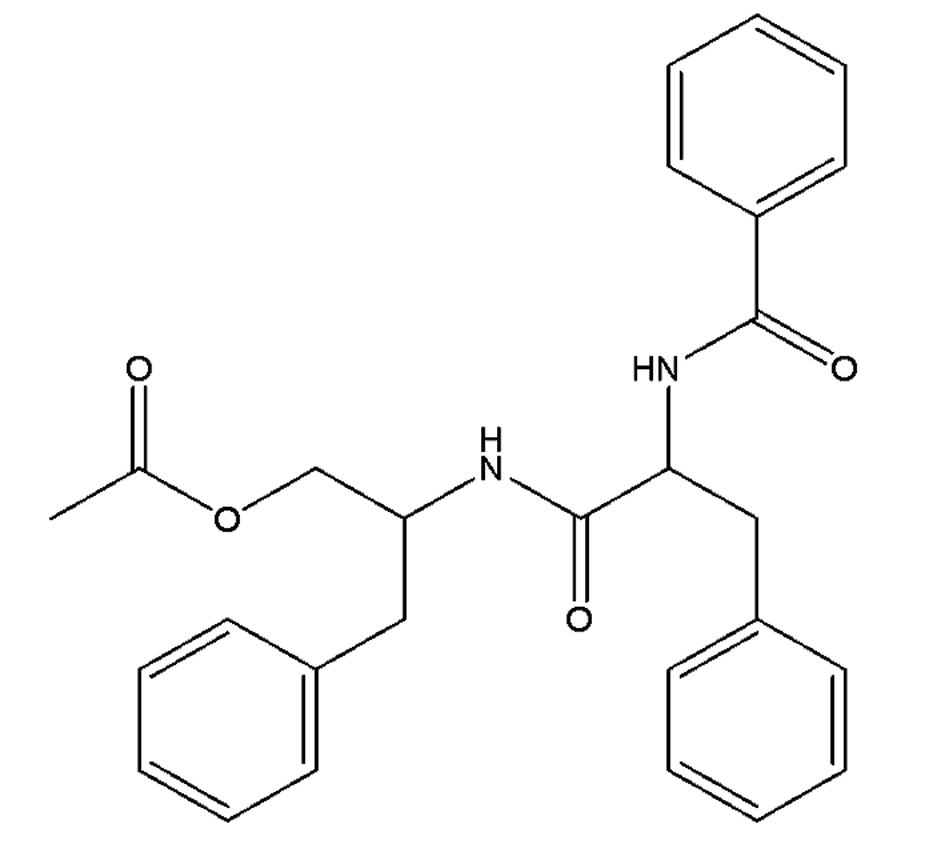

Supplement: Supplementary file 2 [file jcmm0019-1055-sd2.tif]

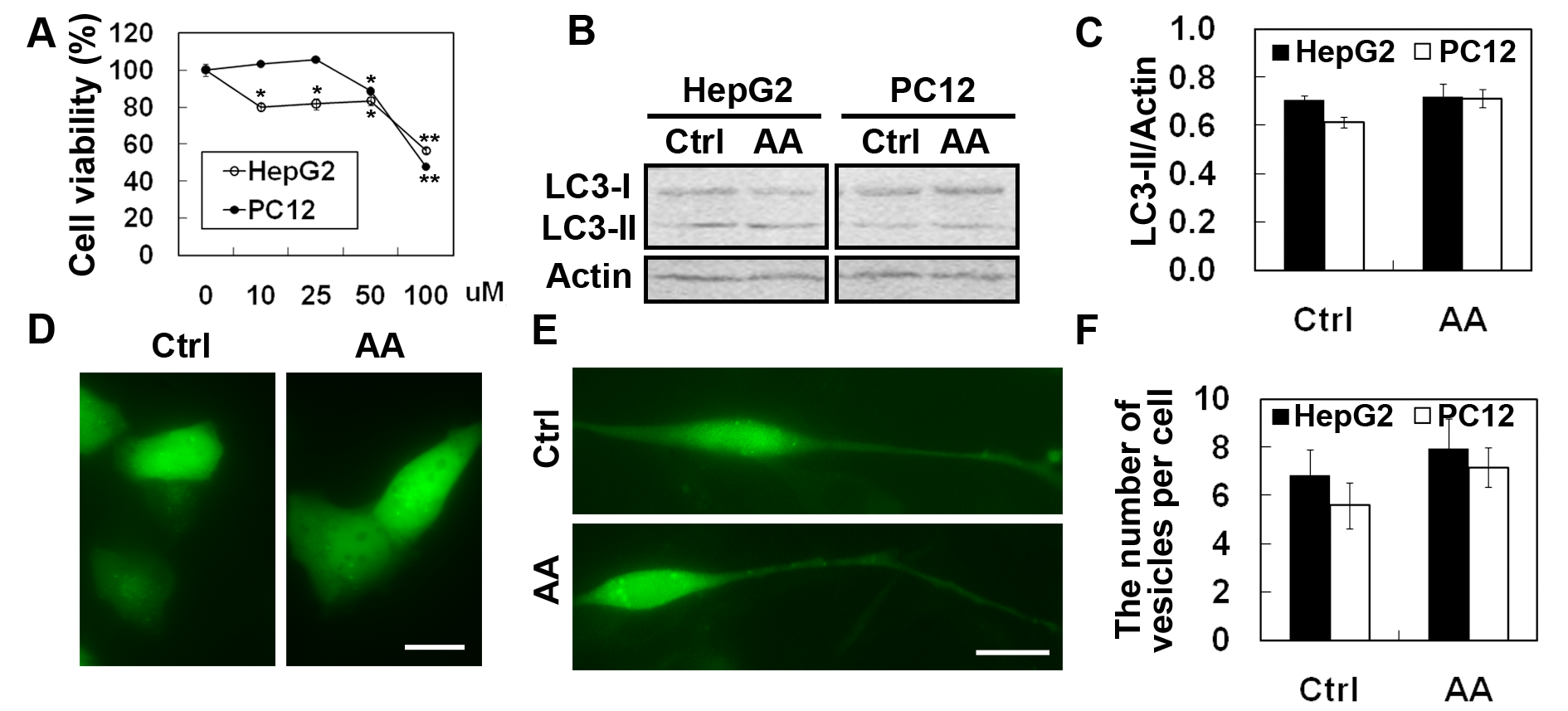

Supplement: Supplementary file 3 [file jcmm0019-1055-sd3.tif]
